# Supplementary material for: Content-rich biological network constructed by mining PubMed abstracts
Source: BMC Bioinformatics. 2004 Oct 8;5:147. doi: 10.1186/1471-2105-5-147 (PMC528731; doi:10.1186/1471-2105-5-147)
Supplement: Additional File 2 — The original results of the above study (non-essential files are deleted to keep the file size under the limit set by BMC bioinformatics). [file 1471-2105-5-147-S2.bz2 › chilibotAdditionalFile2/dip05/216ID7744812E722/html/GRB2_DAG1.html]

 


 **GRB2** and **DAG1** 
  
Found 7 abstracts in PubMed, retrieved 05.  
 

 What does Google say? 
 PDF only 
| .edu only 

---

**Interactive relationship** (e.g. stimulation, inhibition, etc)

**Neutral relationship**- Analysis of the data obtained from SPR reveals a high affinity interaction K D approximately 240 nM between  **Grb2**  and the last 20 amino acids of the beta dystroglycan  [ **DAG1** ]  carboxyl terminus, which also contains a dystrophin binding site.  Ref: 10903901 Biochem Biophys Res Commun, 2000
- The  **Grb2**  beta dystroglycan  [ **DAG1** ]  interaction was inhibited by dystrophin in a range of concentration of 160 400 nM.  Ref: 10903901 Biochem Biophys Res Commun, 2000
- Characterization of the beta dystroglycan  [ **DAG1** ]  growth factor receptor 2  **Grb2**  interaction.  Ref: 10903901 Biochem Biophys Res Commun, 2000
- This complex, in addition to beta dystroglycan  [ **DAG1** ] , was also found to contain  **Grb2**  and focal adhesion kinase p125FAK FAK.  Ref: 10098873 J Neurochem, 1999
- **Grb2** , an adaptor protein involved in signal transduction and cytoskeleton organization, has been shown to bind beta dystroglycan  [ **DAG1** ] .  Ref: 10098873 J Neurochem, 1999

**Non-interactive relationship** (e.g. studied together, co-existance, homology, etc.)

- Both  **Grb2**  SH3 domains bind beta dystroglycan  [ **DAG1** ]  but the N terminal SH3 domain binds with an affinity approximately fourfold higher than that of the C terminal SH3 domain.  Ref: 10903901 Biochem Biophys Res Commun, 2000
- We used surface plasmon resonance SPR, fluorescence analysis, and solid phase binding assay to measure the affinity constants between  **Grb2**  and the beta dystroglycan  [ **DAG1** ]  cytoplasmic tail.  Ref: 10903901 Biochem Biophys Res Commun, 2000
- Together, the data suggest that DA activated MAPK ERK via Ras, Raf 1 kinase, and DAG  [ **DAG1** ]  dependent PKC isoenzymes, but, importantly and contrary to the classical model, this pathway did not involve the  **Grb2**  Sos complex formation.  Ref: 11943676 Am J Physiol Lung Cell Mol Physiol, 2002
- The beta dystroglycan  [ **DAG1** ]   **Grb2**  interaction was investigated and a proline rich region within beta dystroglycan  [ **DAG1** ]  that binds  **Grb2**  src homology 3 domains identified.  Ref: 10903901 Biochem Biophys Res Commun, 2000
- Blot overlay experiments demonstrate that alpha dystroglycan  [ **DAG1** ] , beta dystroglycan  [ **DAG1** ] , and syntrophins all bind  **Grb2** , the growth factor receptor bound adapter protein.  Ref: 11551227 Biochemistry, 2001
- We isolated both FAK and  **Grb2**  from synaptosomal extracts by chromatography on immobilized recombinant beta dystroglycan  [ **DAG1** ] .  Ref: 10098873 J Neurochem, 1999
- At the synapses, the adaptor protein  **Grb2**  may mediate FAK beta dystroglycan  [ **DAG1** ]  interaction, and it may play a role in transferring information between the dystroglycan  [ **DAG1** ]  complex and other signaling pathways.  Ref: 10098873 J Neurochem, 1999
